# MicroRNA-15a/β1,4-GalT-I axis contributes to cartilage degeneration via NF-κB signaling in osteoarthritis

**DOI:** 10.1016/j.clinsp.2023.100254

**Published:** 2023-07-19

**Authors:** Hairong Wang, Weilin Wang, Jian Wang, Linsheng Zhang, Yujie Luo, Xiaobo Tang

**Affiliations:** Department of Orthopedics, Jianhu People's Hospital, Yancheng, Jiangsu, China

**Keywords:** MicroRNA-15a, β1,4-GalT-I, Cartilage degeneration, NF-κB, Osteoarthritis

## Abstract

**Objective:**

Osteoarthritis is a condition characterized by articular cartilage degradation. The increased expression of β1,4-Galactosyltransferase-I (β1,4-GalT-I) in the articular cartilage of osteoarthritis patients was related to an inflammatory response. The aim of this study was to elucidate the role of β1,4-GalT-I in osteoarthritis. This study aimed to determine the function of 1,4-GalT-I in osteoarthritis.

**Methods:**

The osteoarthritis mouse model with the destabilization of the medial meniscus was established by microsurgical technique. Pathological changes in articular cartilage were observed by hematoxylin and eosin staining and safranin O-fast green staining. Quantitative real-time polymerase chain reaction, western blot, and enzyme-linked immunosorbent assays were used to observe mRNA and protein expression, respectively. RNA interactions were verified by a luciferase reporter assay. SA-β-Gal staining was used to assess chondrocyte senescence. Immunofluorescence staining was conducted to observe the localization of Nuclear Factor-kappaB (NF-κB).

**Results:**

β1,4-GalT-I and microRNA-15a (miR-15a) show high and low expression in the articular cartilage of osteoarthritis, respectively. MiR-15a inhibits the mRNA translation of β1,4-GalT-I. β1,4-GalT-I promotes extracellular matrix degradation, senescence, and NF-κB activation in IL-1β-stimulated chondrocytes, which can be reversed by overexpression of miR-15a. Intra-articular injection of microRNA-15a ameliorates cartilage degeneration by inhibiting β1,4-GalT-I and phosphorylation of NF-κB *in vivo*.

**Conclusion:**

The authors clarified that the miR-15a/β1,4-GalT-I axis inhibits the phosphorylation of NF-κB thereby inhibiting extracellular matrix degradation and senescence in chondrocytes to alleviate cartilage degeneration in osteoarthritis. MiR-15a and β1,4-GalT-I may serve as potentially effective targets for the future treatment of osteoarthritis.

## Highlights

• MiR-15a/β1,4-GalT-I axis inhibits ECM degradation and senescence in chondrocytes.
• MiR-15a inhibits β1,4-GalT-I expression with post-transcriptional modifications.
• β1,4-GalT-I promotes NF-κB phosphorylation to exacerbate osteoarthritis.
• Intra-articular injection of miR-15a attenuates cartilage degeneration *in vivo*.

## Introduction

Aging and long-term mechanical stress, both of which cause articular cartilage deterioration, are the leading causes of Osteoarthritis (OA).^1^ Chondrocytes are the most widely known type of cell found in the cartilage matrix, which is responsible for maintaining the matrix's homeostasis ^2^. A growing body of studies have reported that some genes were dysregulated in chondrocytes, which caused abnormal proliferation and inflammation responses, thereby facilitating cartilage degeneration.^3^,^4^

β1, 4-Galactosyltransferase-I (β1,4-GalT-I) is an essential enzyme in a variety of biological activities, including glucose metabolism.^5^ Immune cell adhesion at the site of inflammation was related to increased β1,4-GalT-I in articular cartilage as well as synovial tissue of OA patients as compared to the sane.^6^ By decreasing Toll-Like Receptor 4 (TLR4) signaling and p65 and IKK phosphorylation, β1,4-GalT-I has also been demonstrated to diminish joint inflammation and prevent chondrocyte apoptosis in OA.^7^ According to previous research,^8^ β1,4-GalT-I is significantly elevated in the synovial tissue of rheumatoid arthritis patients, which is involved in the inflammatory response of synovial tissue. Furthermore, in a surgically induced OA model, the expression of β1,4-GalT-I was found to be increased on the first day after the operation.^9^ These findings suggest that β1,4-GalT-I plays a crucial role in the development and progression of inflammation in the synovial tissue associated with OA.

MicroRNAs (miRNAs) are one type of epigenetic modulator that can regulate the protein levels of the targeted mRNAs.^10^ Recent studies have progressively confirmed miRNAs can be combined with the 3’-Untranslated Region (UTR) of targeted mRNA to result in the irreversible degradation of mRNA, thereby regulating the development and progression of diverse diseases, including OA.^11^ Over the past decade, multiple miRNAs have been implicated in OA progression by the involvement of the degradation of Extracellular Matrix (ECM) and cartilage degeneration.^12^

For example, by inhibiting the nuclear factor-erythroid 2-related factor 2 as well as the antioxidant capacity of chondrocytes, miR-146a promotes cartilage degeneration.^13^ Through suppression of the Nuclear Factor-kappaB (NF-κΒ) signaling, inhibition of miR-140 and miR-146a reduced the inflammatory response to OA.^14^ Moreover, miR-26a reduced articular cartilage damage and synovial inflammation in OA by inhibiting the NF-Κβ.^15^ However, the exact miRNAs that directly regulate β1,4-GalT-I during OA development remain elusive.

In the present study, the authors found that miR-15a was a direct regulator of β1, 4-GalT-I in osteoarthritic chondrocytes and uncovered the role of miR-15a/β1, 4-GalT-I axis in cartilage degeneration based on *in vitro* and *in vivo* analysis. The present results clarify that the miR-15a/β1, 4-GalT-I axis inhibits NF-κB phosphorylation to reduce ECM degradation and senescence in chondrocytes, thereby ameliorating cartilage degeneration in OA. Future treatments for OA could focus on miR-15a and β1, 4-GalT-I as possible therapeutic targets.

## Materials & methods

### Animal model establishment and intervention

A total of 36 male C57BL/6 mice that were specifically pathogen-free and weighed 28±2g and 10 weeks old were acquired from the Animal Experiment Center of Jiangsu University. At the animal experiment center, all of the mice were housed in an area free of specific pathogens and given unfettered access to food and water. The temperature was kept at 25 °C, and the relative humidity ranged from 50 to 60 percent. Microsurgical techniques were utilized in order to develop a mouse model of OA that included a Destabilization of the Medial Meniscus (DMM), with reference to previously published material.^16^ Twelve mice, six in each of the Sham and DMM groups, were euthanized six weeks after surgery to acquire joint and tissue samples. There were six mice in each of the following groups: Sham, DMM, DMM + Lentivirus (Lv)-NC, and DMM + Lv-miR-15a. Then, a week after surgery, each group underwent weekly intra-articular injections of 0.9% NaCl (10 μL), 0.9% NaCl (10 μL), Lv-NC (20 mmoL/L, 10 μL), and Lv-miR-15a (20 mmoL/L, 10 μL), respectively. The experiments involving animals in this study strictly followed the ARRIVE guidelines.^17^ Every procedure was approved by the Animal Care and Use Committee of Jianhu People's Hospital (protocol n° 2021JH009).

### Cell culture and treatment *in vitro*

Merck Millipore (California, USA) supplied the human normal chondrocyte cell line (C28/I2). At a situation of 37 °C as well as 5% carbon dioxide, cells were cultured in Dulbecco's modified Eagle's medium/F-12 (Gibco, USA) supplemented with 10% fetal bovine serum (Gibco, USA) and added with 1% streptomycin and penicillin (Gibco, USA). IL-1β (Proteintech, USA) at 10 ng/mL was employed to stimulate chondrocytes in order to produce a mimetic OA model *in vitro*. Chondrocytes cultivated in vitro were separated into five groups: IL-1β, miR-15a mimic + IL-1β, β1,4-GalT-1 OE + IL-1β, IL-1β + miR-15a mimic + β1,4-GalT-1 OE and control group. Following transfection, chondrocytes in the group that contained IL-1β were subjected to stimulation with IL-1β (10 ng/mL) for 24 h, whereas the control group was grown under conditions without IL-1β for one day. Then, each group's cells were utilized for subsequent tests.

### Transfection

The miR-15a mimics, the β1,4-GalT-I overexpression vector, the Negative Control (NC)-Lentivirus (Lv-NC), and the overexpression miR-15a-Lentivirus (Lv-miR-15a) used in the present study were afforded by the RiboBio biotechnology company (Guangdong province, Guangzhou, China). In accordance with what is outlined in the manual provided by the manufacturer, chondrocytes were effectively transfected with miR-15a mimics and 1,4-GalT-I overexpression vectors at an ultimate dose at 50 nM, employing Lipofectamine 3000 reagent (Thermo Fisher Scientific, USA).

### Histopathological analysis

After soaking for 24 h in 4% paraformaldehyde, knee tissue from a mouse was decalcified by soaking it for 20 days in a 10% Ethylene Diamine Tetraacetic Acid (EDTA) solution. After decalcification was complete, the samples were cleaned with distilled water before being examined, and then immersed for 8 hours in 70, 80, 90, 95 and 100% ethanol to dehydrate. Paraffin embedding was then performed. The samples were cut to 4 μm thickness. Following the articulated directions in the instructions offered by the manufacturer, the sections were dewaxed as well as hydrated prior to being stained with safranin O-fast green staining and Haematoxylin and Eosin (H&E) staining. For the purpose of determining the level of damage to cartilage tissue, the Osteoarthritis Research Society International (OARSI) scoring criteria were applied.

### Quantitative real-time polymerase chain reaction (qPCR)

The Trizol reagent (Thermo Fisher Scientific, USA) was used to successfully extract RNA after a cartilage tissue was ground with liquid nitrogen. Utilizing the FastKing cDNA 1^st^ Strand Synthesis Kit (Tengen, KR116-01), RNA was converted into cDNA from 1g of total RNA in a reverse transcription reaction. PCR was carried out using cDNA serving as the template, and the process was conducted in accordance with the guidelines offered in the TaKaRa SYBR Premix Ex Taq II kit. The amplification conditions were 95 °C for 4 min, 94 °C for 30 s, 62 °C for 40 s, 72 °C for 45 s, and the above for 35 cycles, and finally at 72 °C for 10 minutes. During the final cycle, amplification curves, lysis curves, and CT values were collected. This study used GAPDH as an internal reference gene. The calculation of the gene expression was carried out using the 2^−ΔΔCt^ method.^18^ Supplementary Table S1 displays the primer sequences used in this experiment.

### Western blot

The grinding technique with liquid nitrogen was used to accomplish the procedure of tissue lysis on the knee cartilage. Chondrocytes were lysed using RIPA lysis solution. The total concentration of protein in each sample was calculated using a BCA kit. Then protein samples were denatured in water at 100 °C for 5 min. After the proteins had been denatured, 30 µg each protein sample was processed through an SDS-PAGE gel electrophoresis. Subsequently, proteins in SDS-PAGE gel are transferred to the PVDF membrane by electrophoresis. Electrophoresis conditions were set at 80V for 20 minutes, followed by 1 hour at 120V. Protein transfer to PVDF membranes was done at 120V for 1.5 h. PVDF membranes were sealed with skimmed milk at a solution of 5% for one hour at ambient temperature. After completion of membrane washing with TBST solution, the membranes were incubated with primary antibodies at 4 °C overnight. The membrane was then washed with TBST solution. Upon completion of the washing of the membranes with the TBST solution, membranes were incubated with the primary antibodies for a period of one night at 4 °C. After that, in order to eliminate proteins that did not stick to the membrane, it was washed on the shaker with TBST solution. The membranes were incubated with corresponding Horseradish Peroxidase (HRP) ‒ conjugated secondary antibodies for 2 h at ambient temperature. Upon washing the membrane with the TBST, it was color developed by an ECL reagent. Subsequently, a ChemiDoc Touch (Bio-Rad, USA) was used to capture the images. Bands were analyzed for grey scale values using Image J software. β-actin was used as the inner control of the protein expression. Supplementary Table 2 contains information about the antibodies.

### Prediction of target sites by TargetScan

Referring to previously published literature,^19^ the authors were able to estimate the targeted site of miR-15a at 1,4-GalT-I by using the TargetScan website, which can be accessed online at https://www.targetscan.org.

### Dual-luciferase constructs and reporter assay

The binding of β1,4-GalT-I gene to the miR-15a was *in vitro* elucidated by a luciferase reporter assay. The 3′-UTR sequence in β1,4-GalT-I was obtained using the NCBI database. The corresponding primer sequences were synthesized, and the recombinant plasmids were constructed by Guangzhou Ribo Bioengineering Company. The 3′-UTR sequence in β1,4-GalT-I gene was successfully amplified by PCR and ligated using GV272 as a vector to construct the wild-type GV272-β1,4-GalT-I-WT 3′-UTR and mutant GV272-β1,4-GalT-I-MUT 3′-UTR of the recombinant plasmids. Then, Negative Control (NC) and miR-15a mimics were synchronously transfected with these two recombinant plasmids into HEK293T cells, respectively, according to the directions for the X-tremeGENE HP DNA Transfection Reagent (CAS: 06366244001, Roche, Switzerland). In order to determine the level of luciferase activity present in the samples, the Luciferase Assay Reagent II and the Stop & Glo® reagent were added to them in accordance with the protocols outlined in the Dual-Luciferase® Reporter Assay System (Promega, USA, CAS: e1910). The absorbance was measured on a Multiskan FC ELISA (Thermo, USA).

The pGL3-NF-κB-Pro reporter gene recombinant plasmid was purchased from Guangzhou Ribo Bioengineering Company. Transfection of plasmids into chondrocytes was conducted, using the X-tremeGENE HP DNA Transfection Reagent (CAS: 06366244001, Roche, Switzerland). The luciferase activity was then tested as described above.

### Enzyme-linked immunosorbent assays (ELISA)

The supernatant was collected following the *in vitro* cultivation of chondrocytes, and then it was centrifuged at a temperature of 4°C for 5 minutes at a speed of 1500 rpm. The collected supernatant after centrifugation was used for protein concentration assays. The High Mobility Group Protein B1 (HMGB-1) ELISA Kit (FineTest, cat.no EM0382), as well as the TNF-α ELISA Kit (FineTest, cat.no EM0183), were utilized in order to assess the concentrations of soluble HMGB-1 and TNF-α, respectively, in line with the directions that have been supplied by the respective manufacturers. Finally, the absorbance at 450 nm was measured using a Microplate Reader (Reagen, Beijing, China).

### Assay of senescence-associated β-galactosidase (SA-β-Gal) activity

The senescence-associated -galactosidase staining Kit was utilized in order to determine the level of β-galactosidase activity present in vitro cultured chondrocytes. Chondrocytes were cultured in a 6-well cell culture plate (Beygold, China). Cells were gently rinsed once using PBS. After adding 500 μL of a 4% paraformaldehyde solution, the mixture was left to fix at ambient temperature for 5 min. Subsequently, the paraformaldehyde was removed. The cells were then washed two times by PBS. Each well added one milliliter of SA-gal staining solution and was incubated for 12 h at 37 °C. The number of cells that showed a positive reaction to the SA-β-gal stain was counted under an inverted microscope.

### Immunofluorescence

Chondrocytes were stained immunofluorescently to observe NF-B localization. Coverslips made of sterile material were placed in each well of culture plates with 24 wells. Then, chondrocytes in the logarithmic growth phase (the control group, IL-1β group, miR-15a mimic + IL-1β group, β1,4-GalT-1 OE + IL-1β group, IL-1β + miR-15a mimic + β1,4-GalT-1 OE group) were inoculated at 5×10^4^ cells/well in the treated plates and continued to be cultured for 24 h. The cells were prepared by fixing them with 4% paraformaldehyde (Solarbio, China) and then permeabilizing them with 0.3% Triton X-100 reagent (Solarbio, China), respectively. Cells were incubated with 5% BSA for 1 hour. After adding the rabbit anti-human NF-kB p65 polyclonal antibody (Abcam, CAS: ab16502), the mixture was kept in a refrigerator at 4°C for 8 hours. Subsequently, goat anti-rabbit IgG H&L (Alexa Fluor® 488, Abcam, ab150077) and DAPI were incubated for 2 hours and 10 minutes at ambient temperature, protected from light, respectively. Fluorescence was observed, and pictures were taken under an Eclipse E100+ inverted fluorescence microscope (Nikon, Japan).

### Statistical analysis

GraphPad Prism 8.0 was applied throughout the process of statistical analysis.

The results of the experimental data are expressed as the means ± standard deviations. Calculating the statistical significance of the differences between the two groups involved the use of the unpaired two-tailed Student's *t*-test. The One-Way ANOVA approach was utilized to make comparisons between a variety of different groups. If the p-value was lower than 0.05, then the differences were determined to be statistically significant. Correlations between miR-146a, miR-140, miR-26a, miR-15a, miR-9, as well as β1,4-GalT-I were done by Pearson correlation analysis.

## Results

### Expression of β1,4-GalT-I is elevated in the articular cartilage of DMM-induced OA mice

**Fig. 1:**
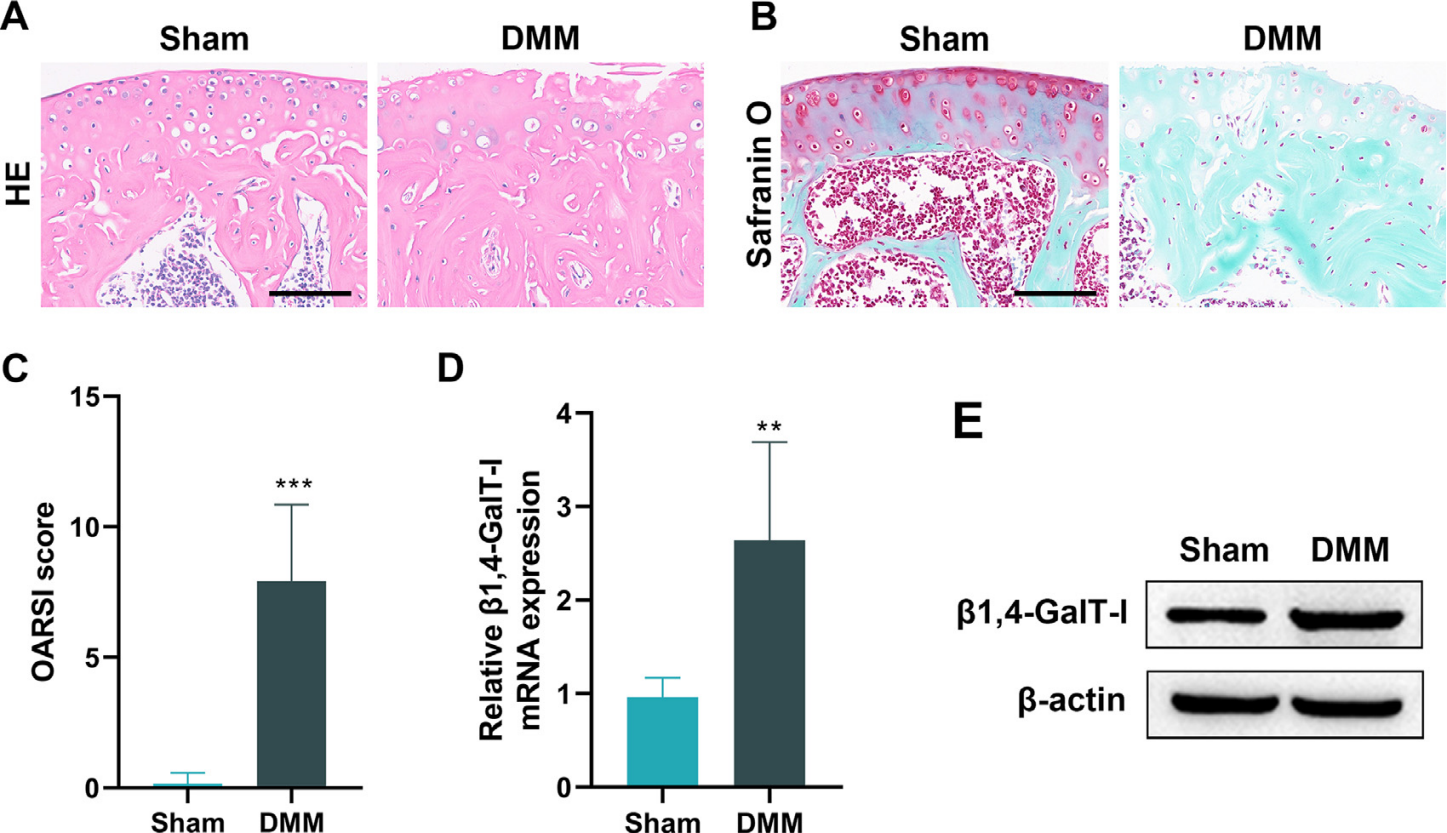
*Expression of β1,4-GalT-I is elevated in the articular cartilage of DMM-induced OA mice*. The mice were randomly assigned to a DMM group and a Sham group, with six mice in each group. (A and B) Representative images of articular cartilage stained with H&E (A) and safranin O-fast green (B) (scale bar: 100 μm). (C) OARSI score of the joints. (D and E) The qPCR and western blot were used to detect mRNA and protein expression of β1,4-GalT-I in articular cartilage (*** p < 0.001, ** p < 0.01).

In this study, the authors successfully established the DMM mouse model of OA using microsurgical techniques. When compared to the Sham group, the results of H&E staining and safranin O-fast green staining demonstrated that the DMM group had significantly reduced cartilage present in the knee and exhibited considerable damage of articular cartilage (Fig. 1A and.B). The OARSI scores of the DMM group were significantly higher than those of the Sham group (Fig. 1C, p < 0.001). When compared to the Sham group, the mRNA and protein expression of β1,4-GalT-I in the articular cartilage of the DMM group were considerably higher than those found in the Sham group (Fig. 1D and E, p < 0.01).

### MicroRNA-15a is down-regulated and negatively correlated with β1,4-GalT-I in articular cartilage of DMM-induced OA mice

**Fig. 2:**
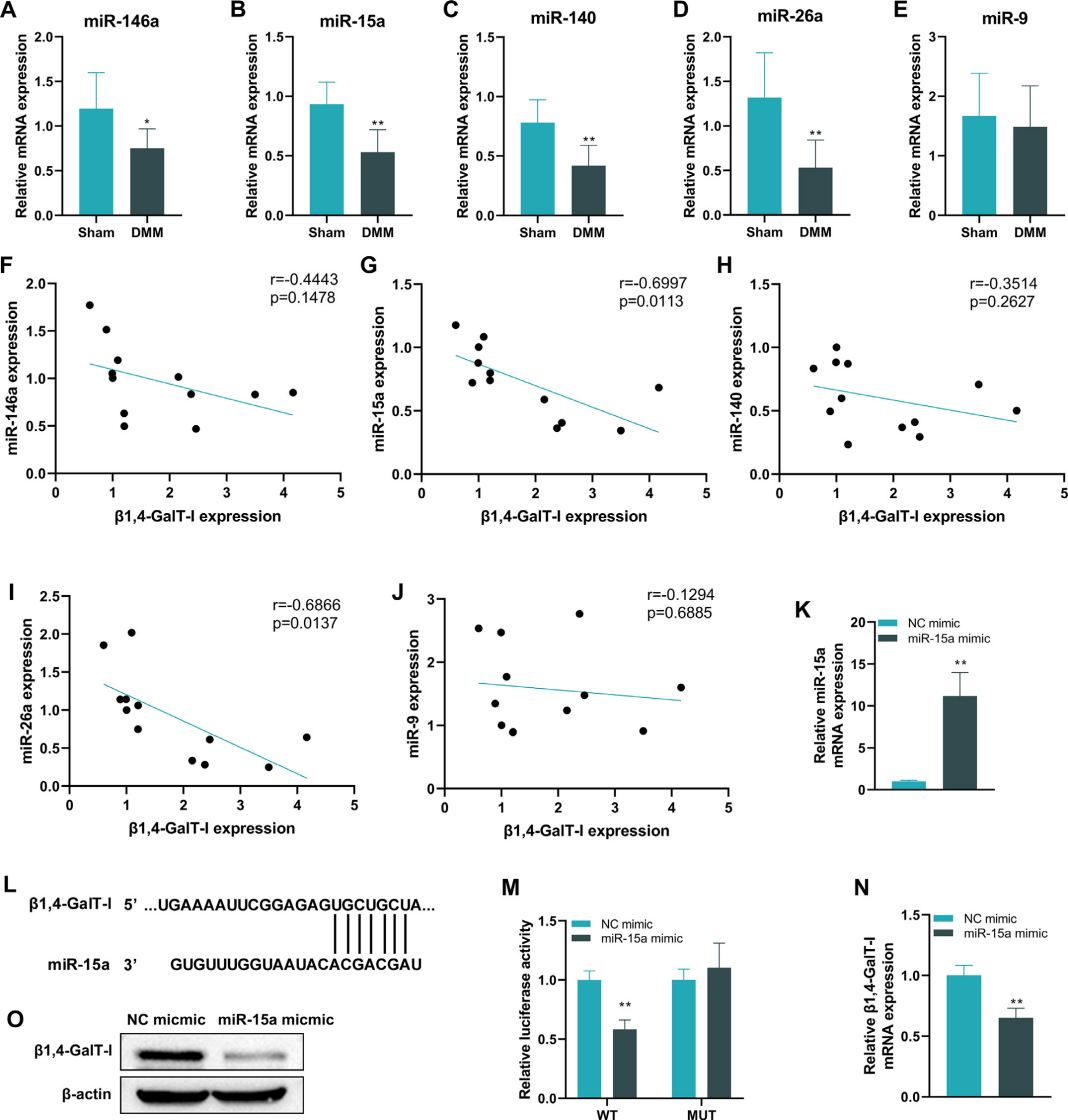
*MiR-15a-5p is down-regulated and negatively correlated with β1,4-GalT-I in articular cartilage of DMM-induced OA mice*. (A‒E) The expression of miR-146a, miR-15a, miR-140, miR-26a, and miR-9 in articular cartilage were detected by qPCR. (F‒J) Pearson correlation coefficient was performed to analyze the correlation of miR-146a, miR-15a, miR-140, miR-26a, and miR-9 with the expression of β1,4-GalT-I. (K) The detection of miR-15a in chondrocytes by qPCR to validate the efficiency of Lipofectamine 3000 transfection of miR-15a mimics into chondrocytes. (L) Prediction of miR-15a targeting sites with mRNA of β1,4-GalT-I by TargetScan. (M) The luciferase reporter assay was conducted to validate the relationship of miR-15a with β1,4-GalT-I. (N‒O) The qPCR and western blot were executed to detect mRNA and protein expression of β1,4-GalT-I in chondrocytes after being transfected with miR-15a mimic, respectively (* p < 0.05, ** p < 0.01).

Through a literature review, the authors collected several miRNAs associated with OA progression, including miR-146a, miR-15a, miR-140, miR-26a, and miR-9. The authors found that miR-146a, miR-15a, miR-140, and miR-26a were reduced in expression in articular cartilage of DMM-induced OA mice, compared with the sham group (Fig. 2A‒E, p < 0.05, p < 0.01, p < 0.01, p < 0.01, no significant difference, respectively). Pearson correlation coefficient analysis showed that miR-15a and miR-26a were negatively correlated with β1,4-GalT-I (Fig. 2G, p = 0.0113, *r* = -0.6997; Fig. 2I, p = 0.0137, *r* = -0.6866). Considering that the correlation between β1,4-GalT-I and miR-15a was greater than that between β1,4-GalT-I and miR-26a, the authors next performed an in-depth study of miR-15a. The authors made chondrocytes stably overexpress miR-15a by transfection (Fig. 2K). The binding site of miR-15a to the 3′UTR region of β1,4-GalT-I was predicted by TargetScan (Fig. 2L). The binding of miR-15a with β1,4-GalT-I was verified by luciferase reporter analysis (Fig. 2M). Moreover, both mRNA and protein expression of β1,4-GalT-I were significantly reduced upon the overexpression of miR-15a (Fig. 2 N‒O, p < 0.01). The above findings show that miR-15a can target and inhibit the transcription of β1,4-GalT-I.

### MiR-15a alleviated ECM degradation and cellular senescence in IL-1β-induced chondrocytes by suppressing β1,4-GalT-I

**Fig. 3:**
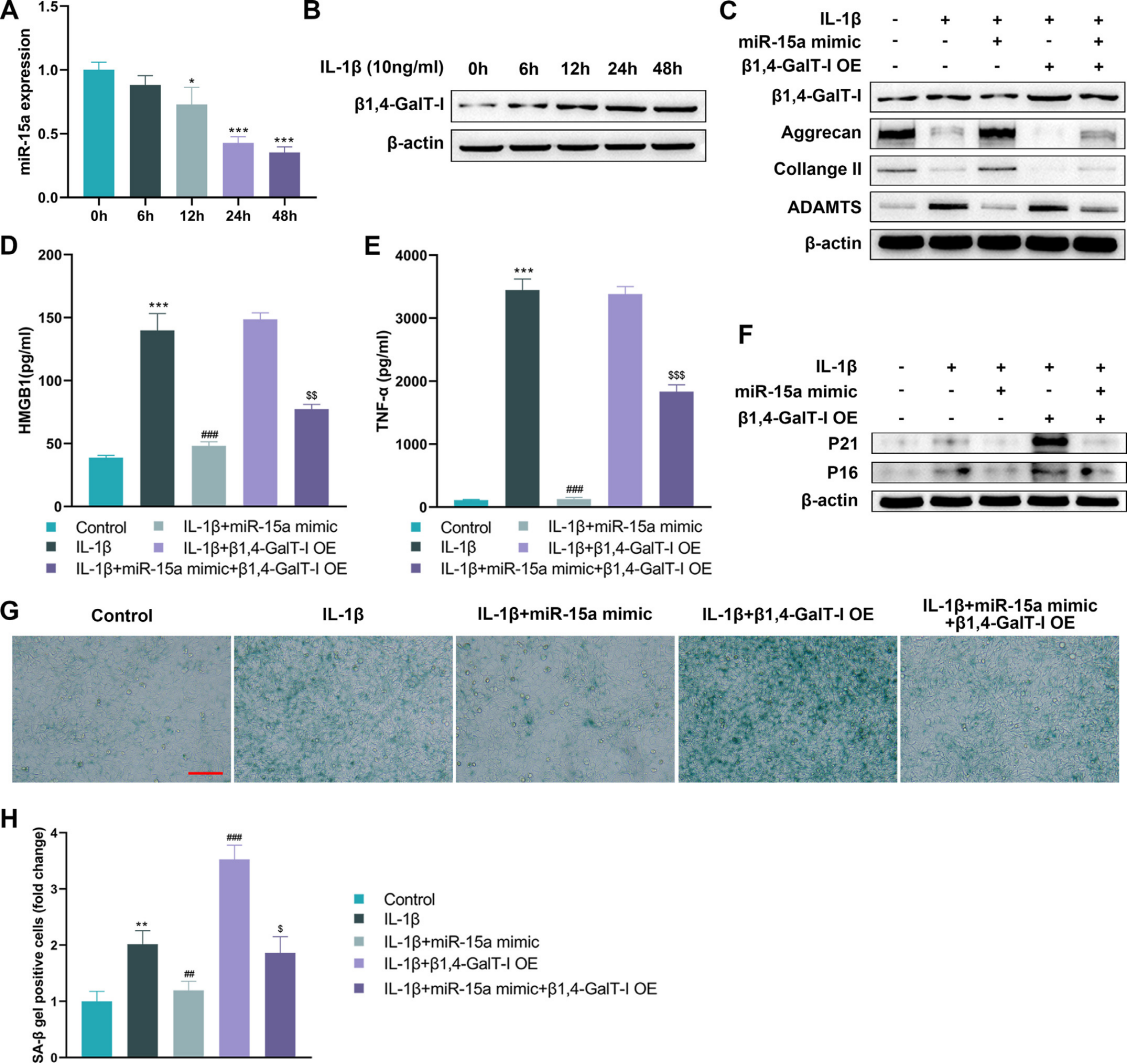
*MiR-15a alleviated ECM degradation and cellular senescence in chondrocytes by suppressing β1,4-GalT-I*. To mimic chondrocytes in OA, the authors stimulated human chondrocytes in vitro with IL-1 (10 ng/mL). The effects of miR-15a and β1,4-GalT-I on extracellular matrix degradation and senescence in chondrocytes was investigated. Chondrocytes *in vitro* were divided into five groups: IL-1β stimulation group, miR-15a mimic + IL-1β group, β1,4-GalT-I overexpression (OE) + IL-1β group, miR-15a mimic + β1,4-GalT-I OE + IL-1β group, and control group. (A and B) The authors observed changes in the expression of miR-15a and β1,4-GalT-I at different stimulation times (0h, 6h, 12h, 24h, and 48h) by qPCR or western blot, respectively (as compared to 0h, * p < 0.05, *** p < 0.001). (C) Western blot was used to detect the protein expression of β1,4-GalT-I, aggrecan, Collange II and ADAMTS5. (D‒E) The Senescence-Associated Secretory Phenotype (SASP), Tumor Necrosis Factor-alpha (TNF-α) and High Mobility Group Box 1 (HMGB1) were detected using ELISA (*** p < 0.001, as compared to control group; ### p < 0.001, as compared to IL-1β group; $$ p < 0.01, $$$ p < 0.001, as compared to IL-1β group). (F) Senescence-related markers (P21 and P16) were detected by western blot. (G‒H) The SA-β-Gal staining of chondrocytes (Scale bar: 100 μm) (** p < 0.01, as compared with control group; ## p < 0.01, ### p < 0.001, as compared with IL-1β group; $ p < 0.05, as compared with IL-1β + miR-15a mimic group).

In order to simulate the chondrocytes found in OA, the authors stimulated *in vitro* chondrocytes with IL-1β. The results showed that miR-15a expression appeared to decrease gradually with prolonged IL-1β stimulation, while protein expression of β1,4-GalT-I increased gradually (Fig. 3 A and B). Previous studies have identified extracellular matrix degradation and chondrocyte senescence as important mechanisms contributing to cartilage degeneration.^20^,^21^ In addition, overexpression of miR-15a inhibited IL-1β-induced expression of the Senescence Associated Secretory Phenotype (SASP) (HMGB1 and TNF-α), and senescence-associated markers (P21 and P16) (Fig. 3 D‒F). SA-β-Gal staining of chondrocytes also showed that the IL-1β+miR-15a mimic group had significantly more SA-β-Gal positive cells than the IL-1β stimulated group (Fig. 3G and H). Notably, the inhibitory effect of miR-15a on IL-1β-induced extracellular matrix reduction and senescence in chondrocytes was counteracted by overexpression of β1,4-GalT-I (Fig. 3C‒H).

### MiR-15a/β1,4-GalT-I axis was involved in regulating NF-κB signaling in IL-1β-induced chondrocyte

**Fig. 4:**
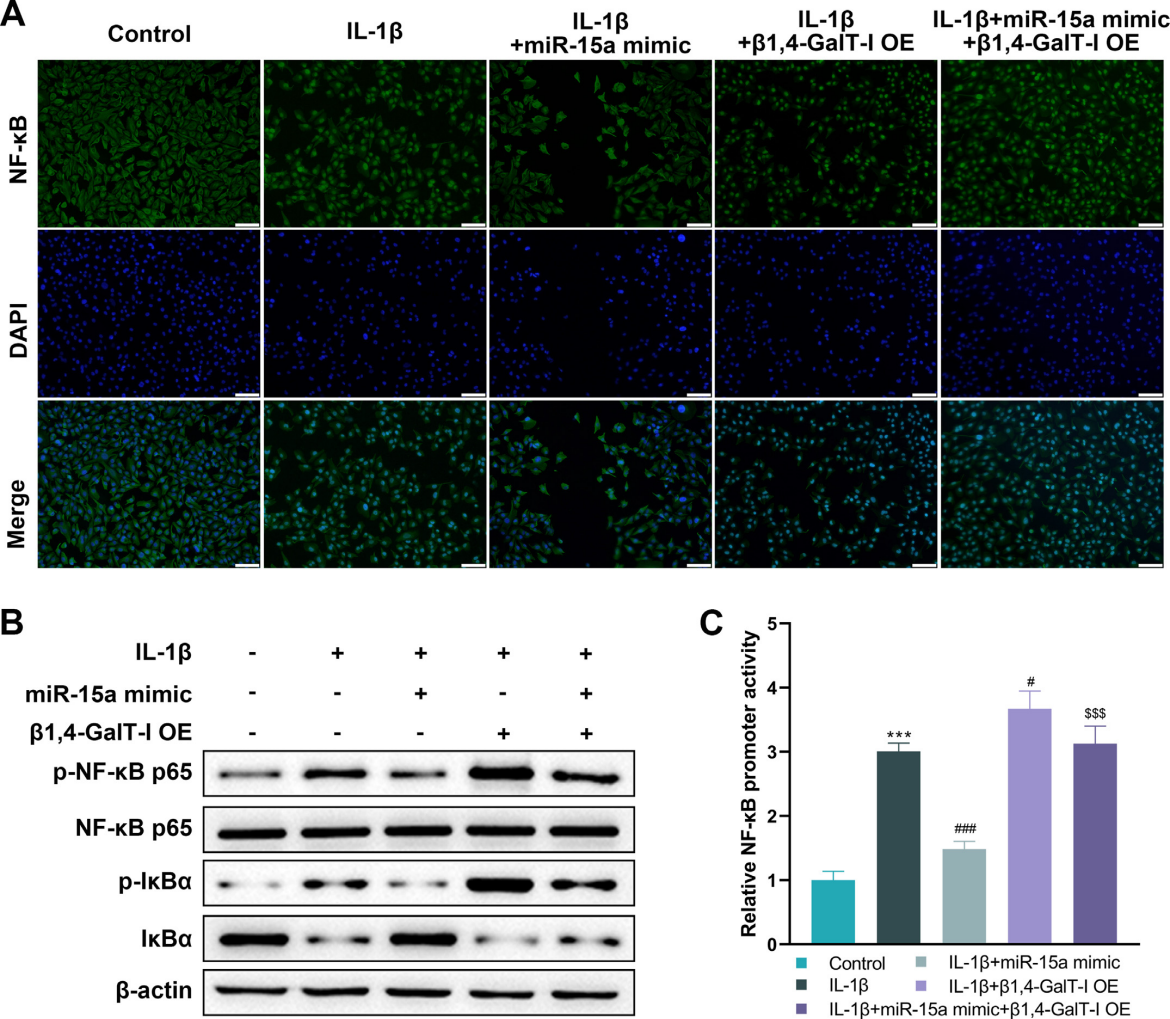
*MiR-15a/β1,4-GalT-I axis was involved in regulating NF-κB signaling in IL-1β-induced chondrocyte*. Chondrocytes cultured in vitro were divided into four groups: IL-1β group, miR-15a mimic + IL-1β group, β1,4-GalT-I OE + IL-1β group, and miR-15a mimic + β1,4-GalT-I OE + IL-1β group (n = 3). (A) The representative images demonstrating the NF-κB in chondrocytes as stained by immunofluorescence. When NF-κB is not activated, it is mainly distributed in the cytoplasm and, when activated, in the nucleus. DAPI shows the location of the nucleus (Scale bar: 100 μm). (B) Western blot was conducted to detect the expression of IκBα, p-IκBα, p-NF-κB p65, and NF-κB p65. (C) The luciferase reporter assay was conducted to detect activity of NF-κB promoter (*** p < 0.01, as compared with the control group; ### p < 0.001, # p < 0.05, as compared with the IL-1β group; $$$ p < 0.001, as compared with the IL-1β + miR-15a mimic group).

In order to find out whether or not NF-κB is activated, the authors observed the localization of NF-κB within chondrocytes through the use of immunofluorescence staining. Under IL-1β stimulation, NF-κB was predominantly distributed in the nuclei of chondrocytes and was enhanced upon overexpression of β1,4-GalT-I. When the miR-15a mimic was transfected into cells, the activation of NF-κB induced by IL-1β was inhibited (Fig. 4A). Subsequently, the authors found that protein expression of both p-NF-κB p65 and p-IkappaB alpha (IκBα) was significantly increased in the presence of IL-1β stimulation and overexpression of β1,4-GalT-I, compared to normal chondrocytes (Fig. 4B). The expression of nuclear factor kappa B p65 did not differ significantly across the groups (Fig. 4B). Next, NF-κB promoter activity was assessed by the luciferase reporter system. The results showed a significant increase in NF-κB promoter activity in chondrocytes under IL-1β stimulation, and a further increase when combined with β1,4-GalT-I overexpression (Fig. 4C). Meanwhile, the transfection of miR-15a mimic effectively blocked the increased NF-κB promoter activity induced by IL-1β, which was reversed by the co-transfection with β1,4-GalT-I overexpression (Fig. 4C).

### Intra-articular Injection of miR-15a ameliorates cartilage degeneration by inhibiting β1,4-GalT-I/NF-κB

**Fig. 5:**
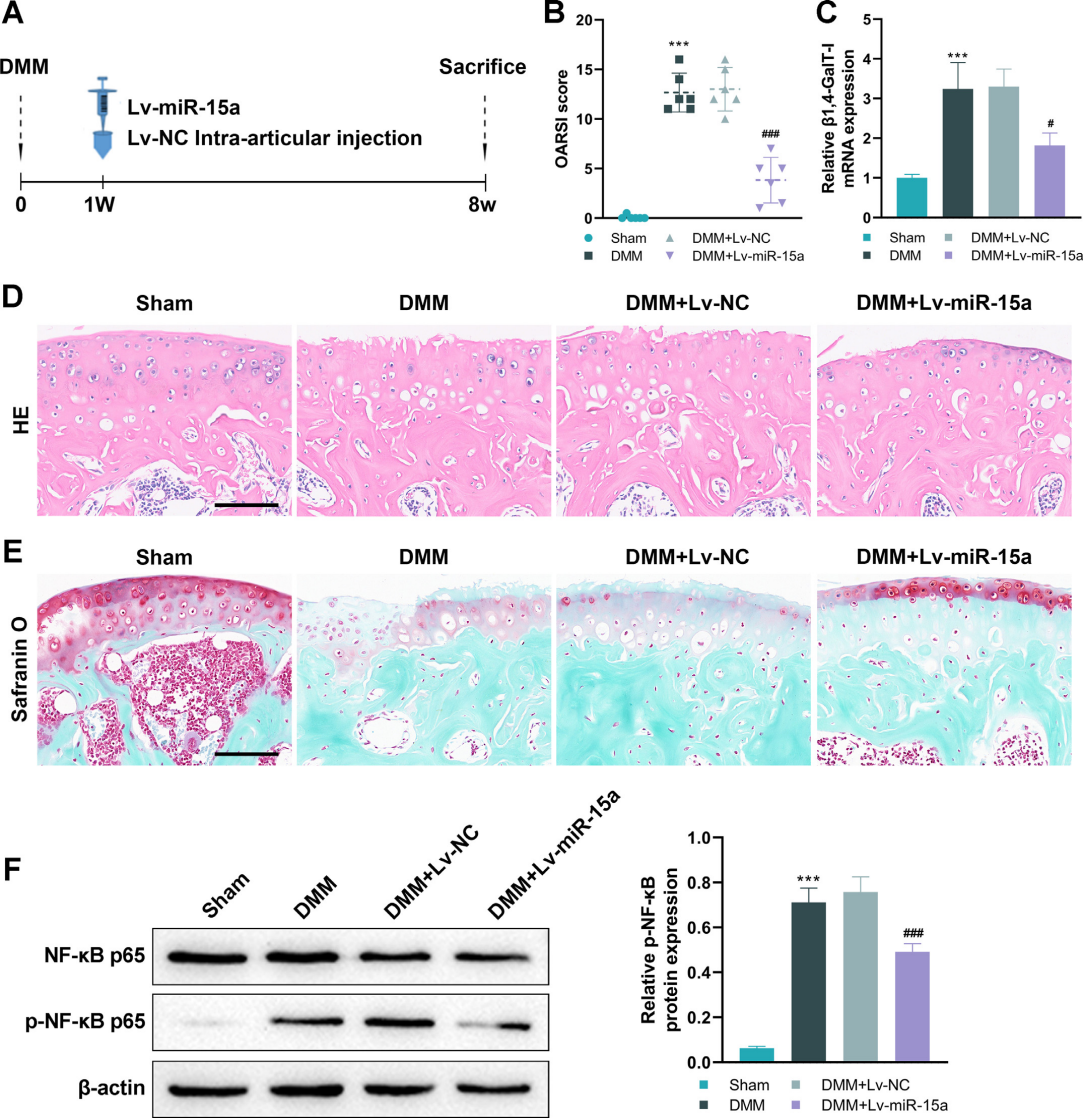
*Intra-Articular Injection (IAJ) of miR-15a ameliorates cartilage degeneration by inhibiting β1,4-GalT-I/NF-Κb*. (A) Experimental layout to observe the effect of miR-15a on cartilage degeneration in vivo. Mice were divided into the Sham, DMM, DMM + Lv-NC, and DMM + Lv-miR-15a groups. The authors established DMM-induced OA mice by microsurgery and administered intra-articular injections of Lv-miR-15a once daily for 7 weeks, one week after surgery. (B) OARSI scores of the joints in each group. (C) The mRNA expression of β1,4-GalT-I in articular cartilage, which was detected by qPCR. (D and E) The H&E staining (D) and the safranin O-fast green staining (E) both showed that the articular cartilage in the knee of the mouse had undergone morphological changes (Scale bar = 100 μm). (F) The NF-κB p65 and p-NF-κB p65 expression in articular cartilage were detected by western blot (# p < 0.05, ### p < 0.001, as compared to DMM group; *** p < 0.001, as compared to Sham group).

To observe the effect of miR-15a on articular cartilage degeneration *in vivo*, the authors constructed DMM-induced OA mice and performed intra-articular injections of Lv-miR-15a one week after surgery (Fig. 5A). The authors found that intra-articular injection of Lv-miR-15a in DMM-induced OA mice significantly reduced OARSI scores (Fig. 5B, p < 0.001) and inhibited mRNA expression of β1,4-GalT-I in articular cartilage (Fig. 5C, p < 0.05), compared to the DMM-induced OA mice group. The H&E staining and safranin O-fast green staining of the joints showed that the destruction and degeneration of articular cartilage in DMM-induced OA mice were significantly inhibited after intra-articular injection of Lv-miR-15a (Fig. 5). Consistent with the *in vitro* IL-1β stimulation of chondrocytes, p-NF-κB p65 was significantly increased in DMM-induced OA mice, which was reversed by intra-articular injection of Lv-miR-15a (Fig. 5F).

## Discussion

In OA, the function and mechanism of β1,4-GalT-I in cartilage degeneration are poorly understood. The authors simulated the damage of OA by constructing a microsurgery-induced OA model and an *in vitro* chondrocyte model. Compared to controls, the authors found that β1, 4-GalT-I was upregulated in articular cartilage and IL-1β-stimulated chondrocytes, while the opposite was observed for miR-15a. In IL-1β-stimulated chondrocytes, β1,4-GalT-I promoted extracellular matrix degradation, senescence, and NF-κB activation, which could be reversed by overexpression of miR-15a. Regarding mechanism, miR-15a inhibited β1,4-GalT-I mRNA translation. Intra-articular injections of miR-15a could reduce cartilage degeneration by inhibiting β1,4-GalT-I and NF-κB activation *in vivo*. MiR-15a and β1,4-GalT-I could be potential targets for the treatment of OA in the future.

According to proteomic studies, the endoplasmic reticulum of aging chondrocytes undergoes excessive sulfur oxidation, resulting in an inflammatory response.^22^ Similar to the previous study,^6^ the present data showed that both the mRNA and protein expression levels of β1,4-GalT-I in the OA mouse model were significantly higher than those in the control. To uncover the potential miRNAs targeting β1,4-GalT-I in OA, TargetScan was applied to predict a list of miRNAs based on β1,4-GalT-I as the target mRNA. Among all predicted miRNAs, five miRNAs, which included miR-146a, miR-15a, miR-140, miR-26, and miR-9, were selected for further verification of the interaction with β1,4-GalT-I as they have been implicated in OA progression in previous reports.^15^^,^^23^, ^24^, ^25^, ^26^ Except for miR-9, the other four miRNAs significantly down-regulated in the OA model. More importantly, the present study found that the expression miR-15a and miR-26 displayed a negative correlation with β1,4-GalT-I expression in OA. Since the miR-15a with β1,4-GalT-I displayed the strongest correlation, miR-15a was chosen for further study.

It has been reported that *in vitro* IL-1β stimulation of chondrocytes induced high β1,4-GalT-I's expression as well as simultaneously inflammation in chondrocytes.^27^ Furthermore, IL-1β stimulated endoplasmic reticulum stress and the NLRP3 inflammasome in chondrocytes.^28^ In chondrocytes, both IL-1β and TNF-α can activate the NF-kB signaling, and lead to the production of IL-6, IL-1β, and TNF-α, and, most notably, apoptosis of chondrocytes.^29^ Therefore, the present present study utilized IL-1β to stimulate chondrocytes *in vitro*.

Previous studies have identified multiple mechanisms involved in chondrocyte reduction in OA, including apoptosis, autophagy, ferroptosis, and senescence.^30^, ^31^, ^32^, ^33^ For example, mechanical overloading induces ferroptosis of chondrocytes through the Piezo1 channel of calcium inward flow.^1^ Overexpression of circular RNAs forkhead box O3 promotes extracellular matrix synthesis and reduces chondrocyte apoptosis by inhibiting the PI3K/AKT signaling pathway.^30^,^34^ E3-ubiquitin ligase HECTD1, regulates chondrocyte autophagy by ubiquitinating Rubicon at lysine residue 534.^35^ β1,4-GalT-I protects chondrocytes from apoptosis induced by TNF-α, effectively.^7^ MiR-15a was confirmed to inhibit TLR4/NF-κB signaling to effectively reduce the dysfunction of chondrocytes.^36^ The findings of the latest investigation demonstrated that NF-κB was inhibited by miR-15a, hence inhibiting chondrocyte senescence.^37^ Here, the authors demonstrated that β1,4-GalT-I stimulates NF-κB and IκBα phosphorylation, which in turn causes extracellular matrix degradation and chondrocyte senescence. The present research suggests that the miR-15a may inhibit chondrocyte senescence. In addition, the mechanism by which the miR-15a/β1,4-GalT-I axis regulates chondrocyte senescence has never been studied. According to the results of the present research, β1,4-GalT-I drives cellular senescence in chondrocytes via activating the transcription factor NF-κB, and miR-15a can reverse this effect. This is a novel mechanism by which miR-15a and β1,4-GalT-I contribute to OA.

Notably, miR-15a expression in OA articular cartilage tended to be low compared to healthy controls.^26^,^38^ Studies have shown that miR-15a promotes the polymerization of proteoglycan and collagen by targeting the ADAMTS5 gene 3′-UTR in the articular cartilage of OA patients (Grade III‒IV).^26^ However, it has also been shown that miR-15a expression is elevated in articular cartilage and promotes extracellular matrix degradation in chondrocytes by inhibiting vascular endothelial growth factor a.^39^ Furthermore, miR-15a accelerates chondrocyte degeneration by inhibiting Parathyroid hormone-related protein.^38^

Here, from animal studies, microsurgery-induced OA would be classified as an acute injury-induced inflammation. In contrast, clinical patients with OA and the specimens obtained are mostly in a chronic inflammatory state. The authors speculate that miR-15a and β1,4-GalT-I possess pro-inflammatory or anti-inflammatory effects on chondrocytes at different times of the inflammatory response in OA. In articular cartilage of microsurgery-induced acute injurious OA, miR-15a was low expressed and β1,4-GalT-I were high.^9^ At this point, miR-15a protected chondrocytes from inflammation-induced senescence and damage, whereas β1,4-GalT-I instead promoted chondrocyte senescence and inflammatory responses. When OA progresses to the chronic inflammatory (Grade III‒IV) stage, miR-15a promotes the degeneration of chondrocytes by inhibiting parathyroid hormone-related protein.^38^ And, β1,4-GalT-I's overexpression will activate the MAP Kinase signal pathway, which would then lead to autocrine production of TNF-α, thereby exacerbating the inflammatory response.^40^ As in previous studies, cyclic AMP-responsive element-binding protein H, a regulator of the inflammatory response, was found to have anti-inflammatory capacity during acute injury and pro-inflammatory response during chronic inflammation.^41^ However, considering that the regulatory mechanisms within chondrocytes are complex, this speculation requires further validation at a later stage.

Even though the authors have found the functionality of β1,4-GalT-I and miR-15a presenting to surgery-induced OA as well as the molecular mechanism behind it, more research is still required. Despite this fact, there are certain limitations to this research. First, the processes that underlie the downregulation of miR-15a in OA chondrocytes remain unclear. Besides, the potential correlation between miR-26 and β1,4-GalT-I has also been uncovered in the present study, but further verification and analysis is required to perform in future study.

In conclusion, the present research showed that miR-15a and β1,4-GalT-I are, respectively, down-regulated, and up-regulated in the cartilage from the OA model. The miR-15a was able to suppress the production of β1,4-GalT-I because it bound to its 3′UTR. Moreover, β1,4-GalT-I caused degradation of cartilage as well as chondrocyte senescence by increasing phosphorylation of NF-κB, and miR-15a was able to mitigate these negative effects by acting as an antagonist. In future diagnostic and therapeutic targets for OA, the miR-15a/β1,4-GalT-I axis may be included.

## Funding

This work was supported by the Medical Research Project of Jiangsu Provincial Health Commission (grant number M2020061); Yancheng Medical Science and Technology Development Program (grant number YK2019067) and Research Project of Jianhu Clinical College, Jiangsu Vocational College of Medicine (grant number 20229JH09).

## CRediT authorship contribution statement

**Hairong Wang:** Conceptualization, Data curation, Formal analysis, Writing – original draft, Writing – review & editing. **Weilin Wang:** Data curation, Formal analysis, Investigation. **Jian Wang:** Conceptualization, Formal analysis, Methodology. **Linsheng Zhang:** Data curation, Formal analysis. **Yujie Luo:** Data curation, Formal analysis. **Xiaobo Tang:** Data curation, Formal analysis, Funding acquisition, Methodology.

## Declaration of Competing Interest

The authors declare no conflicts of interest.

## Appendix Supplementary materials

Image, application 1
