# Supplementary material for: MicroRNA-15a/β1,4-GalT-I axis contributes to cartilage degeneration via NF-κB signaling in osteoarthritis
Source: Clinics (Sao Paulo). 2023 Jul 19;78:100254. doi: 10.1016/j.clinsp.2023.100254 (PMC10387577; doi:10.1016/j.clinsp.2023.100254)
Supplement: Supplementary file 1 [file mmc1.docx]

CLINICS-D-23-00125_Supplementary Material

**Supplemental Table 1** The premier for QPCR.

| **Gene** | **Sequence** |  |
| --- | --- | --- |
| β1,4-GalT-I (mouse) | Forward | ATGAGGTTTCGTGAGCAGTTC |
|  | Reverse | AGAGGTAATAGACGAGGGTGAC |
| β1,4-GalT-I (human) | Forward | CCAGGCGGGAGACACTATATT |
|  | Reverse | CACCTGTACGCATTATGGTCAT |
| miR-146a (mouse) | Forward | TGTCAGACCTGTGAAATTCAGTTCT |
|  | Reverse | AGTGCAGGGTCCGAGGTATT |
| miR-15a (mouse) | Forward | CCATACTGTGCTGCCTCAAAATA |
|  | Reverse | AGTGCAGGGTCCGAGGTATT |
| miR-15a (human) | Forward | AAATACAAGGCTGCCTCAAAATA |
|  | Reverse | AGTGCAGGGTCCGAGGTATT |
| miR-140 (mouse) | Forward | TCTACCACAGGGTAGAACCACG |
|  | Reverse | AGTGCAGGGTCCGAGGTATT |
| miR-26a (mouse) | Forward | CTTGCACGGGGACGCG |
|  | Reverse | AGTGCAGGGTCCGAGGTATT |
| miR-9 (mouse) | Forward | TAAAGCTAGATAACCGAAAGTAAAAATA |
|  | Reverse | AGTGCAGGGTCCGAGGTATT |
| GAPDH (mouse) | Forward | AGGTCGGTGTGAACGGATTTG |
|  | Reverse | TGTAGACCATGTAGTTGAGGTCA |
| GAPDH (human) | Forward | GGAGCGAGATCCCTCCAAAAT |
|  | Reverse | GGCTGTTGTCATACTTCTCATGG |

**Supplemental Table 2** The antibodies used in this study.

| **Antibody** | **Manufacturer** | **Cat. Nº** | **Dilution** |
| --- | --- | --- | --- |
| β1,4-GalT-I | Invitrogen | PA5-106617 | 1:1000 |
| Aggrecan | Invitrogen | MA5-42646 | 1:1000 |
| Collange II | Abcam | ab34712 | 1:1000 |
| ADAMTS5 | Abcam | ab41037 | 1:1000 |
| P21 | Abcam | ab109199 | 1:1000 |
| P16 | Abcam | ab51243 | 1:1000 |
| p-NF-κB p65 | Abcam | ab131100 | 1:1000 |
| NF-κB p65 | Abcam | ab16502 | 0.5 µg/mL |
| p-IκBα | Abcam | ab133462 | 1:10000 |
| IκBα | Abcam | ab32518 | 1:1000 |
| β-actin | Abcam | ab8224 | 1 µg/mL |
| Goat Anti-Mouse IgG H&L (HRP) | Abcam | ab6789 | 1:2000 |
| Goat Anti-Rabbit IgG H&L (HRP) | Abcam | ab6721 | 1:2000 |
| Goat Anti-Rabbit IgG H&L (Alexa Fluor^®^ 488) | Abcam | ab150077 | 1:1000 |
